# Influence of Different Wood Chip Extracts Species on Color Changes and Anthocyanin Content in Synthetic Wine Solutions

**DOI:** 10.3390/foods8070254

**Published:** 2019-07-12

**Authors:** António M. Jordão, Virginia Lozano, María L. González-SanJosé

**Affiliations:** 1 Polytechnic Institute of Viseu, Agrarian Higher School, Department of Food Industries, 3500-606 Viseu, Portugal; 2 Chemistry Research Centre (CQ-VR), 5001-801 Vila Real, Portugal; 3 University of Burgos, Department of Food Science and Biotechnology, 09001 Burgos, Spain

**Keywords:** acacia, anthocyanins, cherry, color, synthetic wine, oak, wood extracts

## Abstract

There is restricted knowledge about the potential impact of the use of different wood species on color and anthocyanin changes during the red wine aging process. This lack of knowledge is even greater when no oak wood species are used. Thus, the aim of this study was to carry out a comparative analysis of the impact of wood chip extracts from oak, acacia, and cherry species on the color characteristics and anthocyanins changes using synthetic wine solutions. In this context, several methodologies were used to quantify, color, and anthocyanins changes during the aging time studied. The results indicated that the contact between wood chip extracts and grape skin isolated anthocyanin extracts induced a decrease of color intensity (particularly red color), and the anthocyanin content in the different experimental synthetic wine solutions studied. All chromatic modifications are potentially detected by human eyes because ΔE values were much higher than 3 CIELab units. These tendencies seem to be independent of the wood species used, but are more pronounced for higher contact time between wood chip extracts and anthocyanins. The obtained results may contribute to a better understanding of the chromatic changes of red wines when aged in contact with different wood chip species.

## 1. Introduction

The use of wood during the process of red wine aging is a common practice in most of the world’s wine producing regions. The main purposes of this practice are to enrich the wine with substances released by the wood, promote reactions due to contact with air diffused through the wood pores, and develop certain interactive chemical reactions, and consequently improve the wine’s quality. In fact, during wine aging in contact with oak wood, particularly using oak barrels, beside oxygen permeation, many compounds such as gallic, ellagic acids, and also ellagitannins are extracted into wine and are involved in a great number of reactions with wine phenolic compounds, particularly with anthocyanins modifying their chemical structure. In addition, the oxidation of anthocyanins during wine aging in oak barrels leads to the dismissal of red color and their combination with oak tannins increases their stability and gives red-purple tones [1,2]. Specifically for monomeric anthocyanins, their concentration in red wine declines constantly during the aging process. Thus, there are a series of mechanisms that might be related to such changes, namely, their adsorption by yeasts, precipitation with proteins, polysaccharides, or with condensed tannins, their oxidation, and the progressive formation of more complex anthocyanin derived pigments, such as pyranoanthocyanins and polymeric anthocyanins produced from condensation between anthocyanins and/or flavan-3-ols directly or mediated by aldehydes and some of them are extracted from oak wood [3,4,5].

Therefore, one of the altered parameters during red wine aging in contact with oak wood is the color, which is a very important sensory parameter of red wines. Several authors describe a decrease of anthocyanin content in red wines aged in contact with different oak woods [6,7,8]. According to Barrera-García et al. [9], the potential anthocyanin decrease in red wine aged in contact with oak wood is a consequence of reactions with ellagitannins extracted from the wood. However, other authors reported a positive impact of oak wood aging in the preservation of individual anthocyanins, namely against oxidation and consequently in red wine color [10,11,12,13]. Venturi et al. [14] also reported that the addition of exogenous tannins extracted from oak wood induces a higher protection of wine against the oxidation process during the winemaking. Wine aging in contact with oak wood also plays an important role in the formation of “new” pigments that could improve and maintain red wine color intensity for longer periods [15,16,17,18]. For example, the formation of several oligomeric and polymeric pigments resulting from reactions between malvidin-3-monoglucoside and (+)-catechin mediated by oak derived compounds, such as furfural, methyl-furfural, vannilin, ellagic acid, and ellagitannins [18,19,20,21]. In addition, some other new compounds formed during red wine aging in contact with oak wood, namely oaklins [21] and condensation reaction products obtained between c-glycosidic, ellagitannins, and malvidin-3-monoglucoside [22] also contribute to changes in the color of wines during aging.

The majority of publications concerning the impact of wood on red wine color changes only the mentioned use of oak wood species, but less attention has been directed to other non oak wood species, such as cherry and acacia. These last two wood species, in recent years, have been considered as a possible source of wood for the wine aging. In fact, some works reported the use of acacia and cherry barrels in wine aging [23,24,25,26,27,28,29]. From a sensory point of view, the red wines aged in contact with oak woods showed a tendency for higher aroma scores than the red wines aged in contact with acacia or cherry woods. However, no statistical differences in taste descriptors between red wines aged in contact with oak woods and red wines aged with other non oak wood species, such as acacia and cherry is reported [29]. In addition, several authors [23,28] reported a positive effect of the use of acacia wood using barrels or chips in the sensory characteristics of white wines in comparison with the use of oak wood barrels or chips. However, despite the above-mentioned works demonstrating the value of cherry and acacia woods in cooperage, little information is available about the potential impact of the use of these wood species in the form of chips during the wine aging process specifically on color parameters and individual anthocyanin content changes.

Thus, the main goal of this study was to carry out a comparative study of the impact of oak (French, American, and Iberian species) and non oak wood (acacia and cherry) chips species on the chromatic characteristics of red wine and on the main compounds responsible for the color of these wines—the anthocyanins. To well understand the factors associated with the cited aim, this research was focused on synthetic wine solutions containing grape isolated anthocyanins and several wood chip extracts from different wood species obtained by two extraction times.

## 2. Materials and Methods

### 2.1. Wood Chip Samples

Acacia (*Acacia pseudorobinia*) purchased by SAI company (Paredes, Portugal), cherry (*Prunus avium*), French, and American oak (*Quercus petraea* and *Quercus alba*, respectively) purchased by AEB Bioquímica company (Viseu, Portugal), and Iberian oak from Portugal (*Quercus pyrenaica*) purchased by J.M. Gonçalves company (Palaçoulo, Portugal)**,** were used in this research work.

All wood chips used exhibited a particle size of 8 mm, a medium toasting (20 min at 160–170 °C) and were previously submitted to a natural drying process.

### 2.2. Wood Chip Extracts Preparation

To reproduce extractions conditions similar to those in wine, the different wood chip samples used in this study were macerated in synthetic wine solution (12% alcohol content and containing 2 g/L of tartaric acid and adjusted to pH 3.5) during 15 and 30 days at 14 ± 2 °C, under darkness conditions and stirred daily (under magnetic stirring for 30 min each day). A concentration of 4 g/L of wood chips was used for extracts preparation. At the end of this maceration, the extracts made in duplicate for each wood chip species and obtained after 15 and 30 extraction days were filtered through wool prior to obtaining two different extracts to be used in this study.

### 2.3. Anthocyanin Grape Skin Extracts Preparation

Red grapes cv. Tinto Fino from a vineyard located in La Horra (Burgos, Spain) were used in this study. Grapes were manually harvested and immediately transported to Burgos University, where they were immediately processed. The grapes had the following maturity characteristics: 25 °Brix and titratable acidity of 7.5 g/L tartaric acid. Grape skins, manual separated from the pulp and seeds, were macerated during one week in a model wine solution (12% alcohol content and pH 3.5 with tartaric-tartrate buffer). The solid:liquid proportion used was 1:2 (*w/v*). At the end of the maceration, liquid fraction was separated by filtration through paper filter (Whatman, Merck, Darmstadt, Germany) and then concentrated under vacuum conditions until around the 30% of initial volume. After this, two consecutive liquid/liquid extractions with ethyl-acetate (1:2, *v/v*) were achieved to eliminate other phenolic compounds extracted from grape skins. Finally, a vacuum treatment removed residual ethyl–acetate, and the resulting anthocyanin extracts were used as source of anthocyanins [30].

### 2.4. Red Synthetic Wine Solutions

Six different experimental red synthetic wine solutions were prepared, all of them in duplicate (Table 1). Control red synthetic wine solutions were arranged by mixing one aliquot of anthocyanin extracts with three parts of the wine synthetic solution used to macerate the wood chips. Similarly, wood red synthetic wine solutions containing anthocyanin and each of the chip extracts (1:3, *v/v*) were arranged. All experimental red synthetic wine solutions were kept in darkness at 14 ± 2 °C for 30 days. At each sampling point (after 15 and 30 days), aliquots of each model mixture were taken and analyzed in duplicate.

### 2.5. Total Phenol and Anthocyanin Content

Total phenol content was quantified by the use of Folin-Ciocalteau reagent, using gallic acid as a standard [31]. The results were expressed as gallic acid equivalents. Total anthocyanin content was quantified by measuring the changes of color according to the pH of the medium [32,33]. The results were expressed as malvidin-3-monoglucoside equivalents.

### 2.6. Individual Anthocyanins Analysis

Individual anthocyanins were analyzed by HPLC-DAD (Agilent LC-DAD series 1100, Waldbronn, Germany) in gradient mode using a C_18_ column, (Nova-Pack^®^, 300 mm × 3.9 mm, particle size 4 μm, Waters, Milford, MA, USA) following the method described by Pérez-Magariño and González-Sanjosé [34] and slightly modified before by Pérez-Magariño et al. [35]. Briefly, the solvents were (A) MilliQ water/formic acid (90:10), (B) MilliQ water/formic acid/methanol/acetonitrile (40:40:10:10) and (C) pure methanol. The flow rate was 0.6mL/min. Linear gradients were applied from 10% to 30% of B solvent for 18 min, from 30% to 45% B for 12 min, and from 45% to 80% B for 18 min, and then isocratic conditions for 5 min. Samples were injected directly after filtration through a 0.45 µm pore size membrane. The UV-Vis spectra (from 240 to 600 nm) were recorded for all peaks. Then, the UV-Vis spectra, the relation between absorbance to 313 and 530 nm, and the retention time were considered to identify the anthocyanins quantified [36]. The quantification of the individual anthocyanins was made by the mean of the calibration curve obtained with standard solutions of malvidin-3-monoglucoside chloride (>95% purity, Extra-synthese, Genay, and France).

### 2.7. Chromatic Parameters Evaluation

Color intensity (A420 + A520 + A620) and tonality (A420/A520) was determined using the analytical methodology described by Glories [36], while CIELaB* coordinates L* (%) (lightness), a* (redness), and b* (yellowness) were evaluated according to OIV method [37]. To distinguish the color more accurately, the color difference was also calculated using the following formula: (ΔE = [(ΔL*)^2^ + (Δa*)^2^ + (Δb*)^2^]^1/2^). Color differences can be distinguished by the human eye when the differences between ΔE values are higher or equal to 3 CIELab units [38].

### 2.8. Statistical Analysis

Usual analysis of variance (ANOVA, one-way) and comparison of treatment means were carried out using SPSS version 23.0 (SPSS Inc., Chicago, IL, USA). Statistically significant differences among obtained results were tested using Duncan’s test (α = 0.05 and *n* = 4, duplicate wood extract x duplicate mixture with anthocyanin extract).

## 3. Results and Discussion

According to the main aim of this study, discussion of the obtained results will be focused on the effect of wood extractable components on the chromatic characteristics of red synthetic wine and on the anthocyanins, which are the compounds with more influence on red wine color.

### 3.1. Effect on Chromatic Characteristics of Red Synthetic Wine Solutions

**Figure 1:**
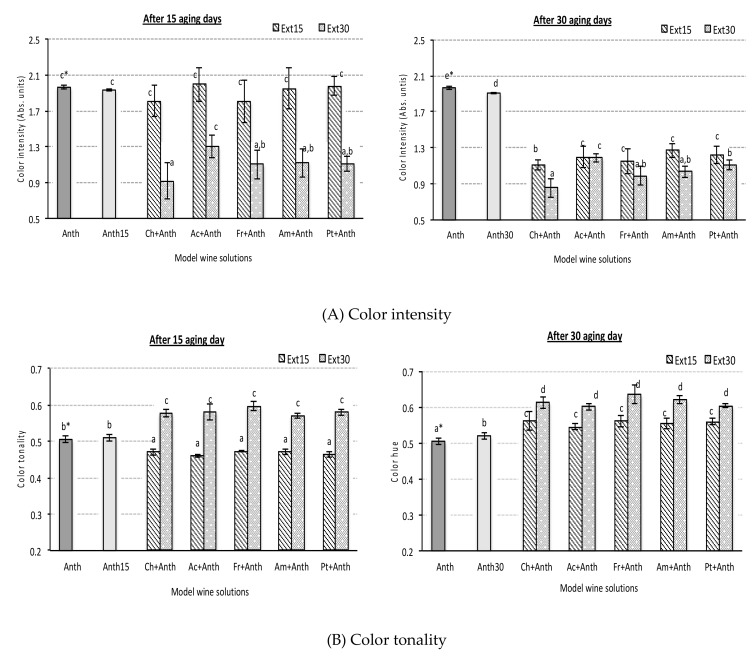
Color intensity (**A**) and tonality (**B**) values in red synthetic wine solutions containing different wood chip species and anthocyanin grape skin extracts after 15 and 30 aging days (sample codes see Table 1). All data express the average of four replicates ± standard deviation; * data points showing the same letter are not significantly different (*p* < 0.05).

It is well known that during the storage and aging period, red wine color commonly changes. Thus, a decrease of color intensity occurs together with a color tonality increase. Obtained results of chromatic characteristics agree with these usual changes of wine color (Figure 1 and Table 2). The decrease of color intensity detected in the different experimental synthetic wine solutions was intense in all cases (an average value reduction ranged from 21.7 to 38.3%, respectively, after 15 and 30 aging days), although it was quicker when wood extracts of 30 extraction days were used. In these cases, significant decreases were observed after the first 15 aging days, while extracts of 15 extraction days produced significant changes only after one month of aging (Figure 1A).

Cherry wood extracts seemed to induce a slight decrease in color intensity, although quantitatively, this fact was only statistically significant after 30 aging days (ChExt30 + Anth sample). Several authors [24,39] demonstrated that the use of cherry wood provides an environment favoring oxidative reactions, and therefore increases the red color loss, making it less suitable for longer wine aging stages. In addition, according to the same authors, cherry wood is also characterized by a very low level of ellagitannins, which provides a reduction of the antioxidant protection of anthocyanins providing a higher oxidative environment than other wood species, and consequently, it potentially further reduces color intensity.

With respect to color tonality (Figure 1B), significant and similar increases were detected for all synthetic wine solutions containing the wood extracts with similar extraction time and the anthocyanin extracts. In agreement with the changes in color intensity, tonality increased quicker and more intensively when wood extracts obtained after 30 extraction days were used. In this case, no significant effect of wood species was observed. Tonality results agree with previous data obtained in red wines aging with oak chips [10,24,40].

Regarding to CIELab* parameters (Table 2), lightness (L*) values showed the usual increase tendency, which correspond to color losses, mainly with the reduction of absorbance at 525 nm [41]. Thus, L* values showed the same tendency that color intensity, being the synthetic wine solutions containing wood extracts of 30 extraction days, those that induced quicker L* value changes. In addition, the general decrease in the tendency observed for a* (redness) agree with the observed results for color intensity showed in Figure 1A. The a* values decreases were particularly intense when wood chip extracts were obtained after 30 extraction days were used. In addition, the lower a* values were detected when wood chip extracts from French oak and cherry wood were used. Previously, Jordão et al. [17,18] also reported, for model wine solutions containing malvidin-3-glucoside, a decrease of this anthocyanin and a* values more pronounced when in the presence of oak wood extracts. Furthermore, for b* values (yellowness), in general, an increase of the values was detected. Corresponding with the increase of color tonality, b* values in general increased after mixed anthocyanin and wood extracts (Table 2). This fact pointed out a clear increase of the yellow color that was more intense when extracts of oak wood species (French, American, and Iberian species) were used. Besides this, the results of 15 days of maceration extracts wrote down a significant increase of b* values after the first 15 aging days. This point, together with the stability of a* values, could point out a possible protective color of these extracts, but only during this period. It is important to note that the extraction of several wood phenolic compounds could explain an increase in b* values (yellowness) that were already detected in red and white wines aged in contact with different wood chips [28,29].

Finally, the values obtained for color difference (ΔE) between control and the other synthetic wine solutions showed that, in all cases, ΔE values were much higher than 3 CIELab units (values ranging from 8.0 to 35.4 CIELab units, Table 2) and then all chromatic modifications were potentially detected by human eyes [38]. According to previously commented results, ΔE values showed intense increase after 15 aging days when wood extracts of 30 days of extraction were used, and in general, they had longer aging time and higher values of ΔE were observed.

Considering all chromatic results obtained, it is possible to assert that, as higher levels of extractable wood components (Table 3), higher modification of color was observed, and this fact could have negative consequences on quality, especially due to the drastic reduction of the color intensity. In fact, it was clear that synthetic wine solutions containing extracts obtained with higher extraction time (30 days) showed, in general, a significant increase of total phenolic content.

### 3.2. Effect on Total Anthocyanin and Phenolic Levels

**Figure 2:**
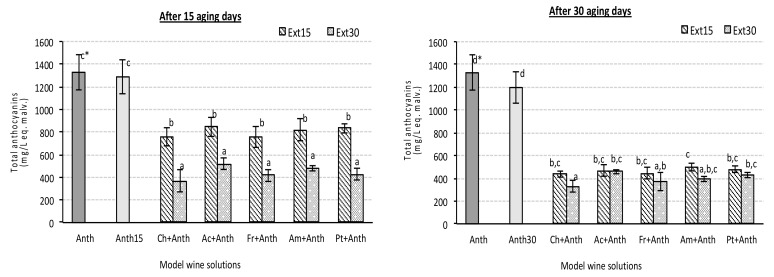
Total anthocyanins quantified in experimental red synthetic wine solutions containing different wood chip extract species and anthocyanin grape skin extracts after 15 and 30 aging days (sample codes see Table 1). All data express the average of four replicates ± standard deviation; * data points showing the same letter are not significantly different (*p* < 0.05).

Results showed an intense and quick reduction of the global level of total anthocyanins in synthetic wines solutions with wood extracts of 30 days of maceration, and similar results were observed until one month of aging of synthetic wines containing wood extracts of 15 days of maceration (Figure 2). These results explain the evident decrease of color intensity and a* values commented previously. Furthermore, it is well correlated with the increase of L* values, since losses of red pigments produce lighter solutions.

No significant differences among wood species were detected in any case, and after 30 days of aging, all the synthetic wines with wood extracts showed similar levels of global anthocyanins, which were drastically lower than levels of the control synthetic wine. These results showed a clear effect of extractable wood components on the modification of the anthocyanin fraction. Therefore, results point out that extractable wood compounds can constitute a destabilizing factor for the anthocyanins, yielding colorless compounds, with lower absorbance to 520 nm (red color) and higher absorbance in the visible region around 400–460 nm, which correspond with yellow-brown tones, justifying the previously commented increase of tonality and b* values. These results are contrary to previous works in which wood contact was described as a stabilizing wine color process [33,40]. However, it is interesting to have in mind that the cited studies were carried out in wines, where many other compounds can interfere the reactions occurring between anthocyanins and wood compounds, and where anthocyanins can be in more stable structures (co-pigmented and condensed forms) than the “free anthocyanins” extracted from red grapes skins.

From the other point of view, results also showed that the effect of extractable wood components seems to be independent of the quantity and type of extractable wood compounds since all the extracts, independent of their global phenolic content (Table 3), produced similar final effect.

Levels of total phenolic of the wood extracts were significantly different with respect to both factors, wood species and maceration time. In general, as the longer the extraction time higher the quantity of total phenols extracted until reaching a maximum, followed by a decreasing of the phenolic content because of their participation in different reactions. Jordão et al. [42] reported, in model wine solutions, that there is an initial period, during which supply of ellagitannins and ellagic acid to the oak wood chips/model wine solutions contact layer is high, due to the solubility of the compounds, and a second period during which there is a clear and continuous decrease in their values. Presumably, this decrease is a consequence of their participation in oxidation reactions, causing them to degrade and consequently leads to a decrease in their values. However, according to our results, the increase ratio of total phenols was very different among wood species. Thus, while American oak extracts showed very low increment with the time of extraction, this was not statistically significant in fact, French oak extracts showed the highest increase around 42%, followed by Acacia extracts (around 28%) and cherry and Portuguese oak extracts with an increment between 15% and 18%, respectively. These results agree with those of previous works that pointed out that each type of wood showed particular extraction kinetics [43]. For example, the anatomical structure of the American oak wood itself, including its porosity, makes ease the extraction of wood components. This fact may explain that most extractable compounds of American oak were extracted during the first 15 days of maceration. Furthermore, results agree with previous studies that reported a variability of total and individual extractable phenolic compounds between oak and other non oak wood species and indicate a higher total phenolic composition of oak woods in comparison with cherry wood [27,28,29,39,44,45]. In addition, the slight influence of the wood species factor on the final levels of total anthocyanins of the synthetic solutions agree with previous work [33] carried out with chips of different types of oak and with diverse toasting degree.

### 3.3. Effect on Individual Anthocyanin Levels

The analysis of the levels of some individual anthocyanins gave more information about the anthocyanin transformation globally tested by the decrease of total anthocyanins levels.

**Figure 3:**
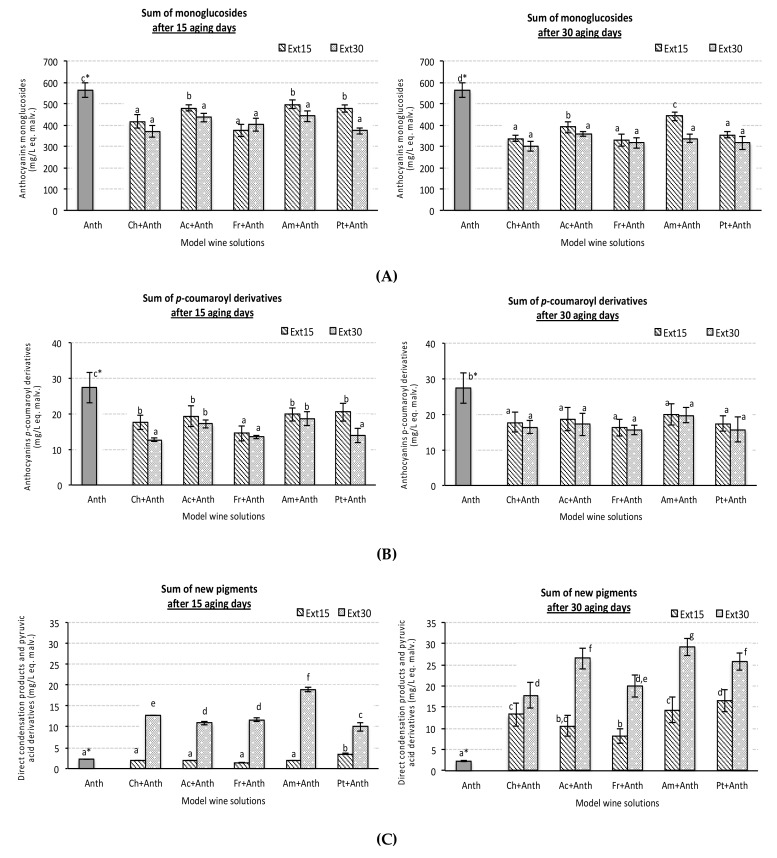
Sum of anthocyanins monoglucosyl derivates (**A**), sum of anthocyanins *p*-coumaroyl derivates (**B**) and sum of new pigments formed (**C**), quantified in experimental red synthetic wine solutions containing different wood chip extract species and anthocyanin grape skin extracts after 15 and 30 aging days (sample codes see Table 1). All data express the average of four replicates ± standard deviation; * data points showing the same letter are not significantly different (*p* < 0.05).

Levels of free monoglucoside anthocyanins, which includes -3-0-glucosyl derivatives of cyanidin, delphinidin, malvidin, peonidin, and petunidin, were significant lower in all synthetic solutions containing wood extracts than in control solution, containing only grape skin anthocyanin extract (Figure 3A). In general, the lowest levels were measured under 30 days of storage. The mean loss of monoglucoside anthocyanins level was around 45%, indicating a drastic reduction of free anthocyanin pigments. Barrera-García et al. [9] also reported 30% lower levels of malvidin-3-monoglucoside after 20 days of contact with wood extracts in model wine solution. No remarkable differences were detected for the extracts with 15 and 30 days of extraction, nor with respect to the wood species factor. These results are contrary to other published works. Thus, Del Álamo Sanza et al. [10,40] reported a greater decrease of monomeric anthocyanins in red wines aged with French than those aged with American oak. In addition, other authors [33] reported a significant effect of oak wood origin on the individual anthocyanin level of a wine macerated with chips, however the effect was variety wood dependent. Once more, the cited differences could be attributed to the effect of other compounds present in wines and none in the synthetic solutions, which can interfere in the reactions that occur between anthocyanins and wood compounds. In addition, it is important to note that some anthocyanins (co-pigmented and condensed forms), formed during fermentation and others during the winemaking process are in more stable structures in wines.

Similar to monoglucoside anthocyanins, levels of the main *p*-coumaroyl derivatives (Figure 3B) were also significantly lower in all synthetic wines containing wood extracts than in the control synthetic wines and no remarkable differences among species were detected. In addition, the decrease ratio of *p*-coumaroyl derivatives was lower than that of monoglucosyl derivatives (30%). These results agree with the higher stability of acyl-anthocyanins with respect to non-acylated derivatives already reported by other authors [46,47,48]. According to Smart [49], wines made from red grapevine cultivars with high proportions of acylated anthocyanins can have greater color stability compared with those from red varieties with no acylated anthocyanins, such as cv Pinot Noir.

New condensed pigments, not present in control synthetic wine containing only grape skin anthocyanin extract, were detected in model wine solutions with wood extracts. Some of them showed retention time and UV-Vis spectrum, like condensed catechin-anthocyanins, while others eluted in time very close to monoglucoside anthocyanins, but showed UV-Vis spectrum clearly different, and in fact allow to different ones to the others. In general, all the new pigments showed UV-Vis spectrum with the maximum absorbance in visible zones lower than 520 nm. None of all the new pigments detected (chromatographic peaks) could be identified and named. Besides this, those “new” chromatographic peaks that were well defined were considered together, being named new pigments group (Figure 3C).

The levels of new pigments were higher when wood extracts of 30 days of extraction were used, and new pigment levels increased along the time of storages. Previously, Jordão et al. [18] reported the formation of new compounds detected in model wine solutions containing malvidin-3-monoglucoside and oak wood extracts after a short storage period. According to these new compounds, it showed a slight increase during 64 storage days.

Significant effects of wood species and maceration time factors on new pigment formation were detected. Wood extracts of 30 days of maceration produced higher and quicker increases than those of 15 days. After 15 days of aging, levels of these pigments were between four and eight times higher than in control wine, and after 30 days of aging, raised increase ranged between 7 and 13 times. The extracts of American oak and 30 extraction days induced the quickest and maximum formation of this type of new pigments, and only synthetic wines with wood extracts of acacia and Portuguese oak, obtained after 30 extraction days, showed levels of new pigments like them.

Observed results could be explained by the ability of anthocyanins to interact with wood components. Among extractable wood components, ellagitannins are easily extracted from wood by water-alcohol and water-acetone mixtures [17,50], and ellagitannins can indeed react with flavanols and anthocyanins to provide condensation products [51,52]. Then, there was a dynamic evolution from several interaction reactions and subsequent transformation of the original pigments results. The loss of free anthocyanins and the new compounds formed contribute to the color differences (ΔE), which were commented previously. Several authors [24,39] reported that the use of cherry wood barrels in wine aging induces a faster evolution of wine pigments with a fast increment of derived and polymeric compounds formation with a consequential decrease of anthocyanin content. However, for the different synthetic wine solutions studied, it was not evident that a more marked increased in new pigments formed in solutions containing cherry wood extracts compared to the others. Other compounds probably different than the anthocyanins themselves may play an important role in new pigments formation. For example, condensed tannins present in wines may help to explain a greater evolution in the formation of new compounds during the wine aging process in contact with the cherry wood, in comparison to the verified one in the synthetic solutions studied.

## 4. Conclusions

The present work points out the impact of the different wood chip species (oak, acacia, and cherry) on anthocyanin content and chromatic characteristics of synthetic wine solutions during the contact time considered. Thus, the obtained results indicated that the interaction of anthocyanins with wood extracted components and the changes of chromatic characteristics derived from these interactions seems to be independent of the wood species.

To the best of our knowledge, this is the first or one of the first researches that has investigated the impact of the use of no oak wood chip species (cherry and acacia) on individual anthocyanin composition and chromatic characteristics of synthetic wine solutions. In this sense, the obtained results may contribute to a better understanding of the chromatic changes of red wines when aged in contact with acacia and cherry wood chips. However, further research will be necessary to improve the knowledge about the potential impact of the use of oak and non oak wood chip species on wine quality.

## Figures and Tables

**Table 1:** Experimental red synthetic wine solutions with different combinations prepared in this study.

Synthetic Wine Solutions	Sample Code
Control wine solution with anthocyanins	Anth
Control wine solution with anthocyanins after 15 aging days	Anth15
Control wine solution with anthocyanins after 30 aging days	Anth30
Cherry wood chip extract ^1^ + anthocyanin grape skin extract	Ch+Anth
Acacia wood chip extract ^1^ + anthocyanin grape skin extract	Ac+Anth
French wood chip extract ^1^ + anthocyanin grape skin extract	Fr+Anth
American wood chip extract ^1^ + anthocyanin grape skin extract	Am+Anth
Portuguese wood chip extract ^1^ + anthocyanin grape skin extract	Pt+Anth

^1^ Synthetic wine solutions containing wood chips extracts obtained after 15 or 30 extraction days (codes = ext15 and ext30, respectively).

**Table 2:** CieLab chromatic coordinates (L*, a* and b*) and color difference (ΔE) quantified in red synthetic wine solutions containing different wood chip species and anthocyanin grape skin extracts after 15 and 30 aging days (sample codes see Table 1).

CIELAB Coordinates	Experimental Synthetic Wine Solutions																				
	Anth	Ch + Anth				Ac + Anth				Fr + Anth				Am + Anth				Pt + Anth			
		15 Aging Days		30 Aging Days		15 Aging Days		30 Aging Days		15 Aging Days		30 Aging Days		15 Aging Days		30 Aging Days		15 Aging Days		30 Aging Days	
		ext15	ext30	ext15	ext30	ext15	ext30	ext15	ext30	ext15	ext30	ext15	ext30	ext15	ext30	ext15	ext30	ext15	ext30	ext15	ext30
L*	53.4 ^a^ ± 3.2	49.1 ^a^ ± 3.6	66.6 ^b^ ± 1.5	82.4 ^c^ ± 3.9	73.8 ^d^ ± 3.6	45.3 ^a^ ± 3.6	64.66 ^b^ ± 2.9	76.3 ^d^ ± 1.9	65.1 ^b^ ± 1.2	49.3 ^a^ ± 4.5	66.2^b^ ± 3.3	79.2 ^c^ ± 2.7	70.8 ^d^ ± 5.3	46.4 ^a^ ± 4.2	62.6 ^b^ ± 2.0	71.9 ^d^ ± 9.6	69.3 ^d^ ± 1.7	45.7 ^a^ ± 1.8	63.9 ^b^ ± 1.7	79.03 ^c^ ± 1.4	67.1 ^d^ ± 1.3
a*	45.5 ^c^ ± 2.8	42.0 ^c^ ± 2.7	23.6 ^a^ ± 1.4	25.4 ^a^ ± 3.1	23.5 ^a^ ± 2.8	45.4^c^ ± 2.8	30.2 ^b^ ± 2.1	32.8 ^b^ ± 2.9	30.2 ^b^ ± 0.8	41.9 ^c^ ± 3.4	23.4^a^ ± 2.4	27.0 ^a^ ± 3.4	23.3 ^a^ ± 3.2	44.2 ^c^ ± 3.2	32.0 ^b^ ± 1.5	29.5 ^b^ ± 3.2	27.1 ^b^ ± 1.3	44.7 ^b^ ± 1.4	28.6 ^b^ ± 1.2	29.4 ^b^ ± 1.6	30.2 ^b^ ± 0.9
b*	−5.7 ^b^ ± 0.8	−12.2 ^a^ ± 1.7	−1.6 ^c^ ± 0.3	−2.1 ^b^ ± 0.1	−0.19 ^d^ ± 0.1	−14.1 ^a^ ± 1.7	−1.5 ^c^ ± 0.2	−3.4 ^b^ ± 0.6	−0.3 ^d^ ± 0.1	−11.6 ^a^ ± 1.7	−0.8 ^d^ ± 0.2	−2.6 ^b^ ± 0.9	0.7 ^d^ ± 0.2	−12.7 ^a^ ± 2.3	−2.0 ^b^ ± 0.4	−2.5 ^b^ ± 1.0	0.6 ^d^ ± 0.1	−13.7 ^a^ ± 0.8	−1.7 ^c^ ± 0.2	−2.3 ^b^ ± 0.5	−0.3 ^d^ ± 0.1
ΔE*	-	8.5 ^a^ ± 0.9	21.4 ^b^ ± 1.3	35.4 ^c^ ± 2.6	30.5 ^c^ ± 2.1	21.3 ^b^ ± 1.9	11.6 ^a^ ± 2.0	26.2 ^b^ ± 3.1	20.8 ^b^ ± 1.1	8.0 ^a^ ± 1.2	21.2^b^ ± 0.5	31.^c^ ± 3.4	28.9 ^c^ ± 1.4	9.9 ^a^ ± 1.0	16.7 ^d^ ± 2.0	25.7 ^b^ ± 2.1	25.0 ^b^ ± 1.1	11.1 ^a^ ± 0.6	18.3 ^d^ ± 0.9	30.4 ^c^ ± 2.9	22.4 ^b^ ± 1.2

L* (%) (lightness); L* (%) (lightness); a* (from green (−) to red (+) ); b* (from blue (−) to yellow (+) ); ΔE* total color difference; the values corresponding to ΔE* were obtained taking as a reference the anthocyanin extract solution alone. Data points derived for each CIELab coordinate in same line showing the same letter are not significantly different (*p* < 0.05). ± Standard deviation. Average values of four replicates.

**Table 3:** Total phenolic content (mg/L expressed in gallic acid equivalents) quantified in synthetic wine solutions containing different wood chip species after 15 and 30 extraction days.

Synthetic Wine Solutions (Wood Chip Species)	Extraction Time	
	15 Days (ext15)	30 Days (ext30)
Cherry	39.91 ^A,a^ ± 0.96	46.82 ^A,b^ ± 1.31
Acacia	40.11 ^A,a^ ± 2.26	51.23 ^B,b^ ± 0.99
French	42.98 ^A,a^ ± 1.31	61.39 ^C,b^ ± 1.70
American	59.85 ^B,a^ ± 1.70	61.58 ^C,a^ ± 1.40
Portuguese	56.40 ^B,a^ ± 1.31	65.03 ^D,b^ ± 0.99

All data express the average of three replicates ± standard deviation. Values with same letter are not significantly different (*p* < 0.05); wherein for same column capital letters are used for wood chips species factor, while for the same line small letters are used for extraction time factor.
